# Supplementary material for: Flame-retardant electrolytes with electrochemically-inert and weakly coordinating dichloroalkane diluents for practical lithium metal batteries
Source: Nat Commun. 2025 Nov 19;16:10188. doi: 10.1038/s41467-025-65138-8 (PMC12630897; doi:10.1038/s41467-025-65138-8)
Supplement: Supplementary file 1 — Supplementary Information [file 41467_2025_65138_MOESM1_ESM.pdf]

Supplementary information for

Flame-retardant electrolytes with electrochemically-inert and weakly coordinating dichloroalkane diluents for practical lithium metal batteries

Zhicheng Wang[1,2‡], Haifeng Tu[3‡], Xingdong Ma[1], Suwan Lu[3], Guirong Su[4], Yiwen Gao[3], Jiangyan Xue[3], Lingwang Liu[3], Xu Yao[1], Kun Liang[1], Ke Wang[5], Fengrui Zhang[1,2], Zhifeng Qin[1], Jieyun Zheng[1,2], Qing Wang[6], Jingjing Xu[3,4,5], Liquan Chen[1,2], Hong Li[1,2,7*], and Xiaodong Wu[1,3,5*]*

[1] Tianmu Lake Institute of Advanced Energy Storage Technologies Co., Ltd., Liyang 213300, P. R. China.
[2] Beijing Advanced Innovation Center for Materials Genome Engineering Key Laboratory for Renewable Energy, Beijing Key Laboratory for New Energy Materials and Devices, Institute of Physics, Chinese Academy of Sciences, Beijing 100190, P. R. China.
[3] *i*-lab, Suzhou Institute of Nano-Tech and Nano-Bionics (SINANO), Chinese Academy of Sciences, Suzhou 215123, P. R. China.
[4] College of Material Science and Engineering, Hohai University, Changzhou 213000, P. R. China.
[5] Changzhou FIRS Battery Technology Co., Ltd., Liyang 213300, P. R. China.
[6] Department of Materials Science and Engineering, National University of Singapore, Singapore 117576, Singapore
[7] Center of Materials Science and Optoelectronics Engineering, University of Chinese Academy of Sciences, Beijing 100049, China.

‡ These authors contributed equally
* These authors jointly supervised this work
Correspondence to: J.X., jjxu2011@sinano.ac.cn, H.L., hli@iphy.ac.cn, X.W., xdwu2011@sinano.ac.cn

Supplementary Table 1 | Physicochemical properties and market price of different diluents.

Solvent	CAS number	M. Wt	Melting point (T_m/℃)	Boiling point (T_b/℃)	Density (g cm^{-3})	Market Price (Adamas, ¥ g^{-1})
Dichloromethane (C1-2Cl)	75-09-2	85	-97	40	1.3	0.36
1,2-Dichloroethane (C2-2Cl)	107-06-2	99	-35	84	1.2	0.29
**1,3-Dichloropropane (C3-2Cl)**	142-28-9	113	-99	120	1.1	0.46
1,4-Dichlorobutane (C4-2Cl)	110-56-5	127	-38	154	1.1	0.32
1,5-Dichloropentane (C5-2Cl)	628-76-2	141	-72	180	1.1	0.59
1,1,2,2-Tetrafluoroethyl-2,2,3,3-tetrafluoropropyl ether (TTE)	16627-68-2	232	-94	92	1.53	3.38

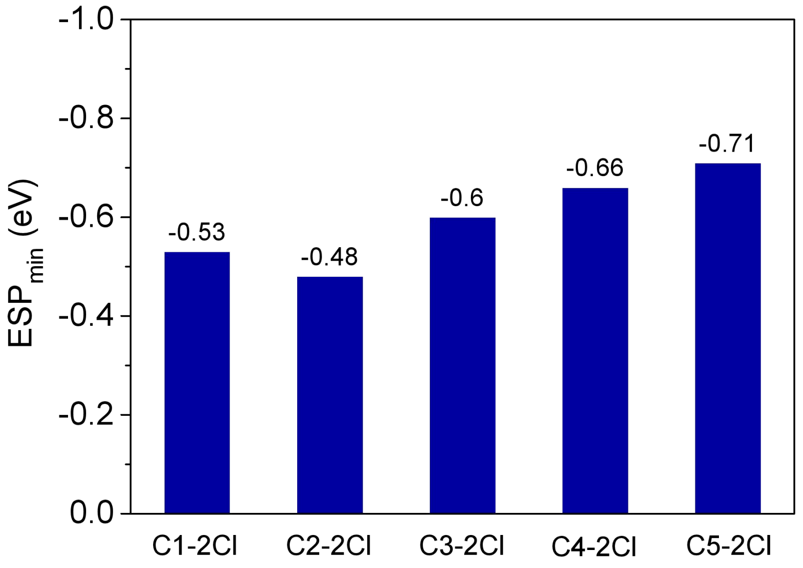

Supplementary Fig. 1 | ESP_{min} of different C-2Cl diluents obtained by DFT calculations.

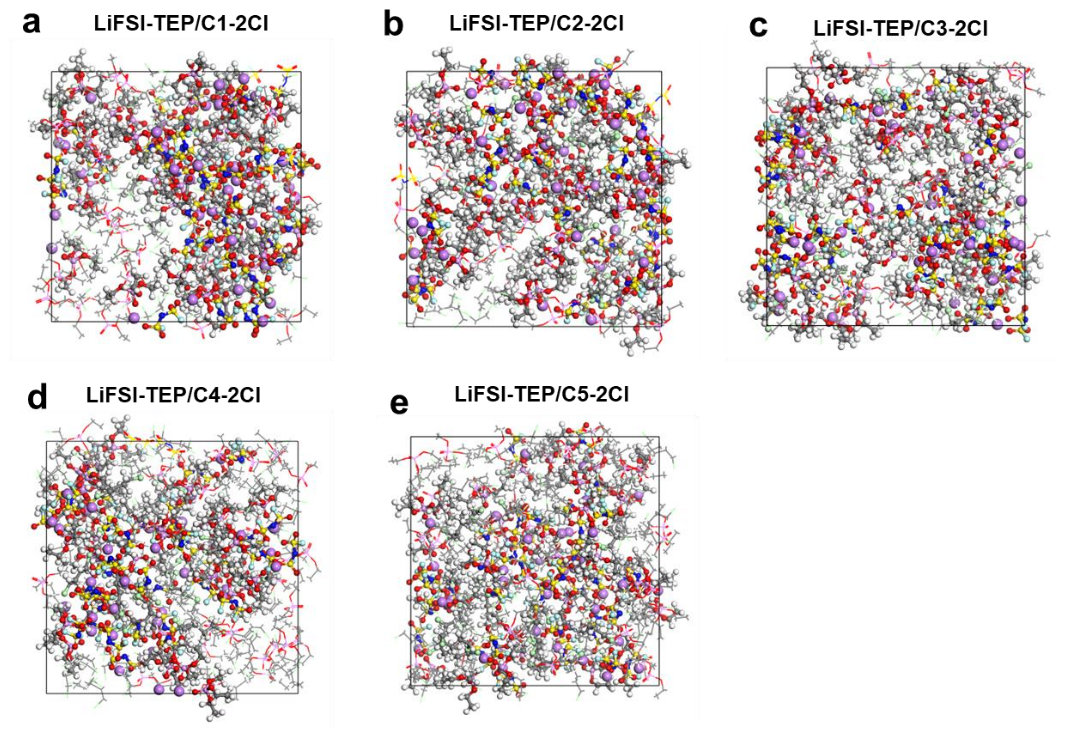

Supplementary Fig. 2 | Snapshots of electrolyte solvation structures with different C-2Cl diluents obtained by MD simulations. **a**, LiFSI-TEP/C1-2Cl. **b**, LiFSI-TEP/C2-2Cl. **c**, LiFSI-TEP/C3-2Cl. **d**, LiFSI-TEP/C4-2Cl. **e**, LiFSI-TEP/C5-2Cl. Li^+ and coordinated anions/solvents/diluents are shown in balls and sticks, free solvents/diluents are shown in lines. Color code: Li-purple, C-dark grey, H-white, O-red, N-blue, S-yellow, F-cyan, P-pink, Cl-green.

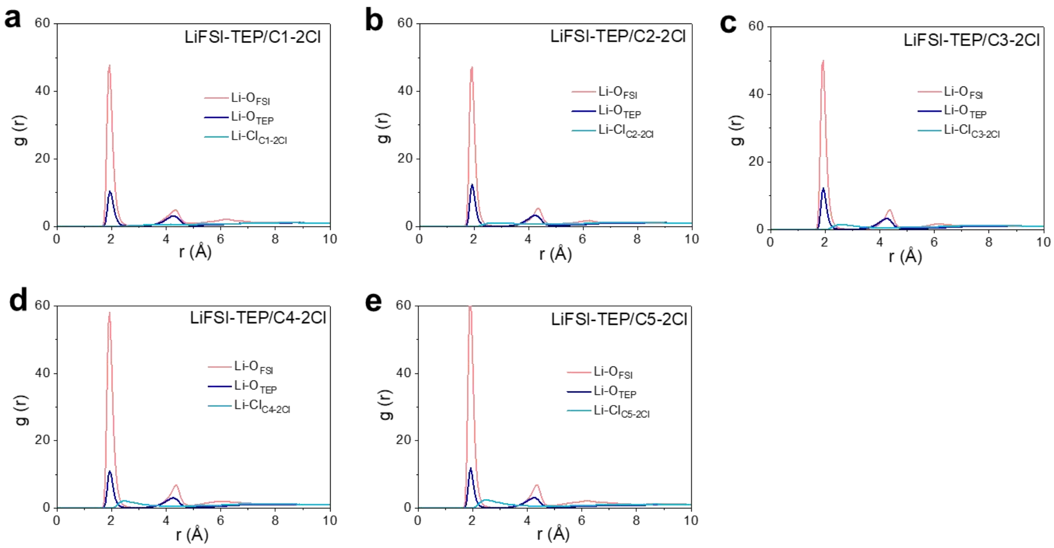

Supplementary Fig. 3 | RDF curves of Li^+ coordination structures in different electrolytes obtained by MD simulations. **a**, LiFSI-TEP/C1-2Cl. **b**, LiFSI-TEP/C2-2Cl. **c**, LiFSI-TEP/C3-2Cl. **d**, LiFSI-TEP/C4-2Cl. **e**, LiFSI-TEP/C5-2Cl.

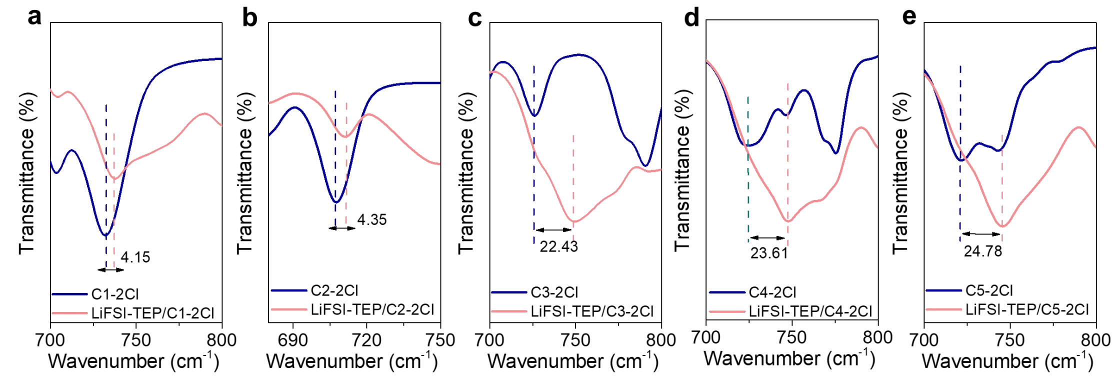

Supplementary Fig. 4 | FTIR curves of different C-2Cl diluents and different electrolytes. **a**, LiFSI-TEP/C1-2Cl. **b**, LiFSI-TEP/C2-2Cl. **c**, LiFSI-TEP/C3-2Cl. **d**, LiFSI-TEP/C4-2Cl. **e**, LiFSI-TEP/C5-2Cl.

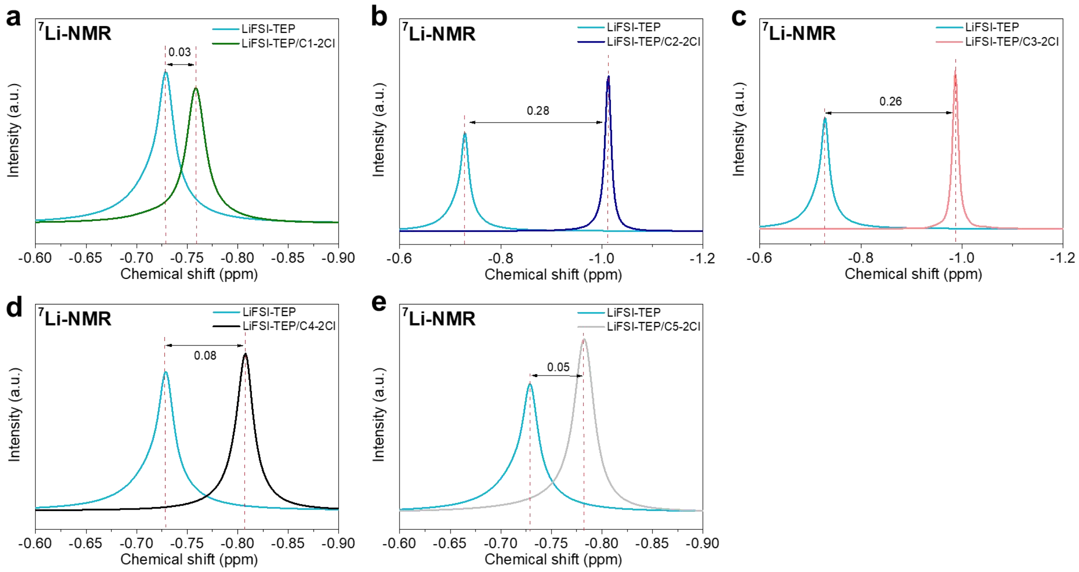

Supplementary Fig. 5 | ^{7}Li NMR curves of different electrolytes. **a**, LiFSI-TEP/C1-2Cl. **b**, LiFSI-TEP/C2-2Cl. **c**, LiFSI-TEP/C3-2Cl. **d**, LiFSI-TEP/C4-2Cl. **e**, LiFSI-TEP/C5-2Cl.

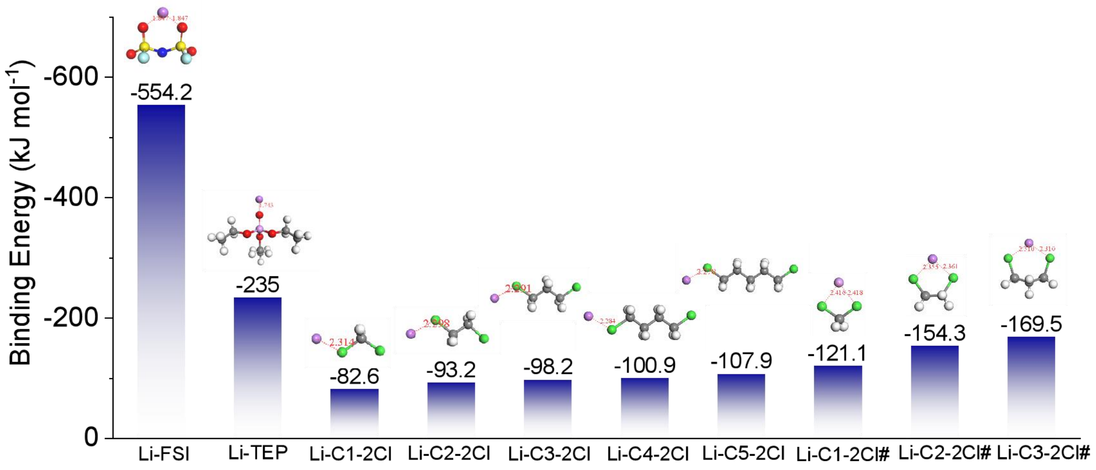

Supplementary Fig. 6 | Binding energies of Li^+ with different anion/solvent/diluents obtained by DFT calculations. Color code: Li-purple, C-dark grey, H-white, O-red, N-blue, S-yellow, F-cyan, P-pink, Cl-green.

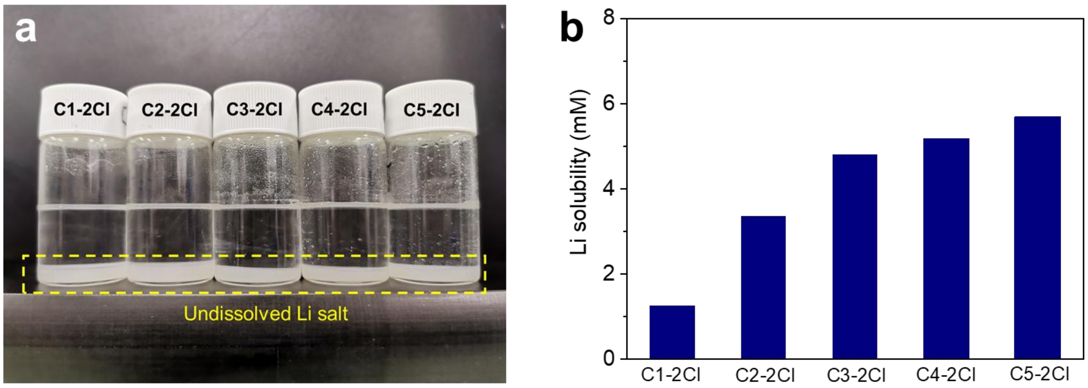

Supplementary Fig. 7 | a, Optical image of 0.1 M LiFSI salt in different C-2Cl diluents after 12 h stirring. **b**, Corresponding Li solubility in different C-2Cl diluents obtained by ICP-OES.

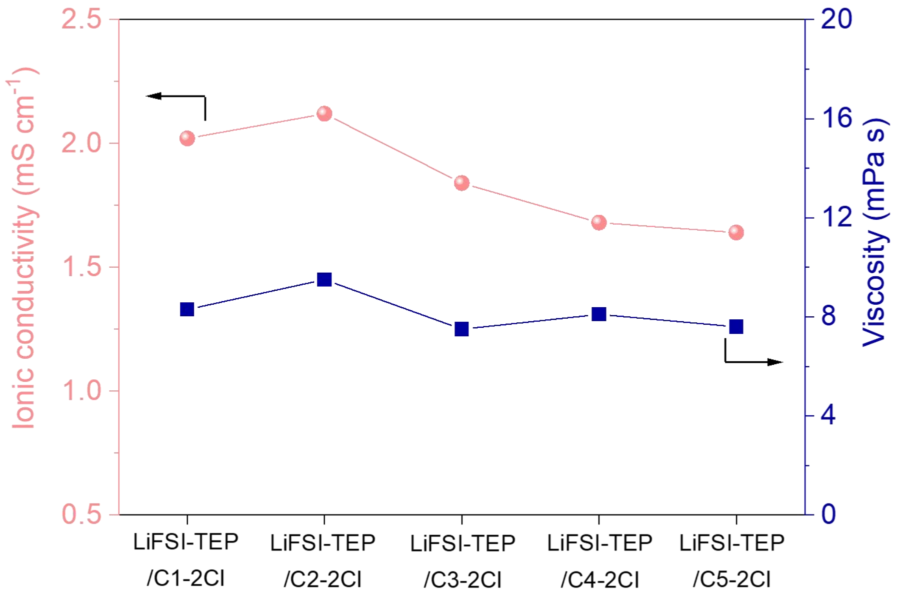

Supplementary Fig. 8 | Ionic conductivity and viscosity of electrolytes with different diluents.

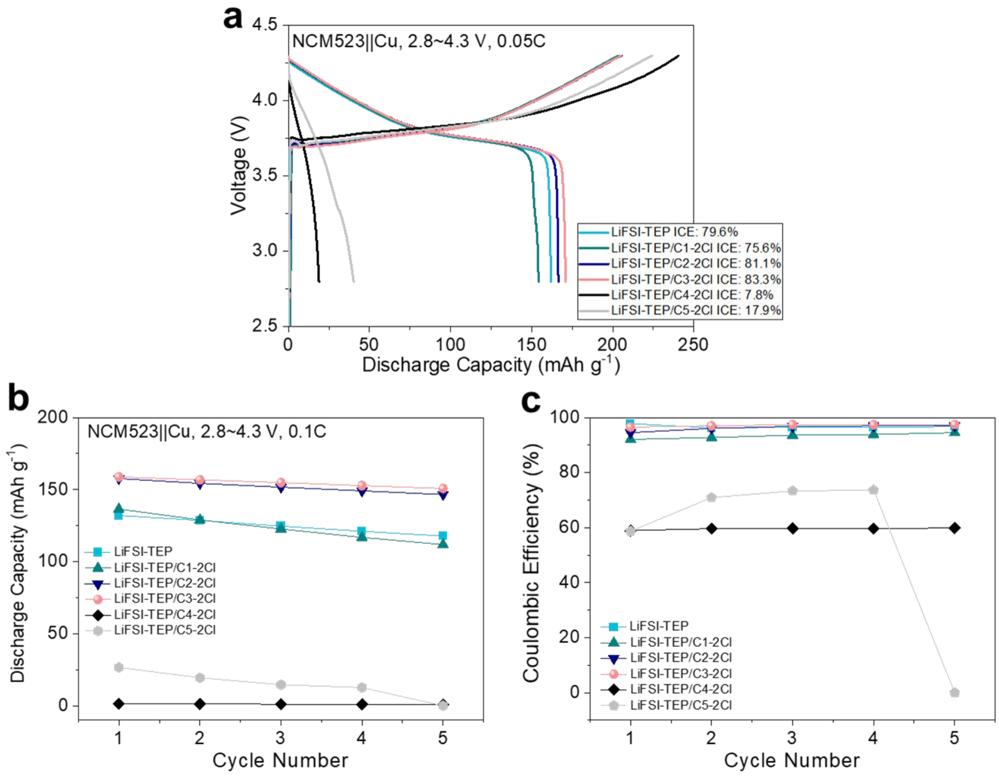

Supplementary Fig. 9 | a, Initial charge-discharge profiles and Initial CEs of Cu||NCM523 cells with different electrolytes at 2.8~4.3 V and 0.05C. **b**, Discharge capacity and **c**, Coulombic efficiency of Cu||NCM523 cells with different electrolytes at 2.8~4.3 V and 0.1C.

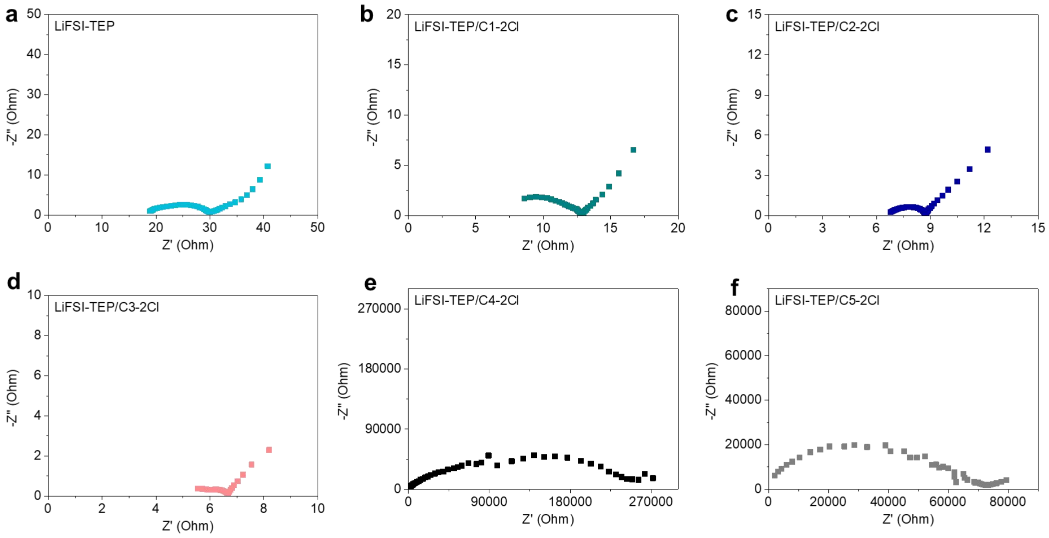

Supplementary Fig. 10 | EIS plots of Li||Li symmetric cells in different electrolytes after 50 cycles. **a**, LiFSI-TEP. **b**, LiFSI-TEP/C1-2Cl. **c**, LiFSI-TEP/C2-2Cl. **d**, LiFSI-TEP/C3-2Cl. **e**, LiFSI-TEP/C4-2Cl. **f**, LiFSI-TEP/C5-2Cl.

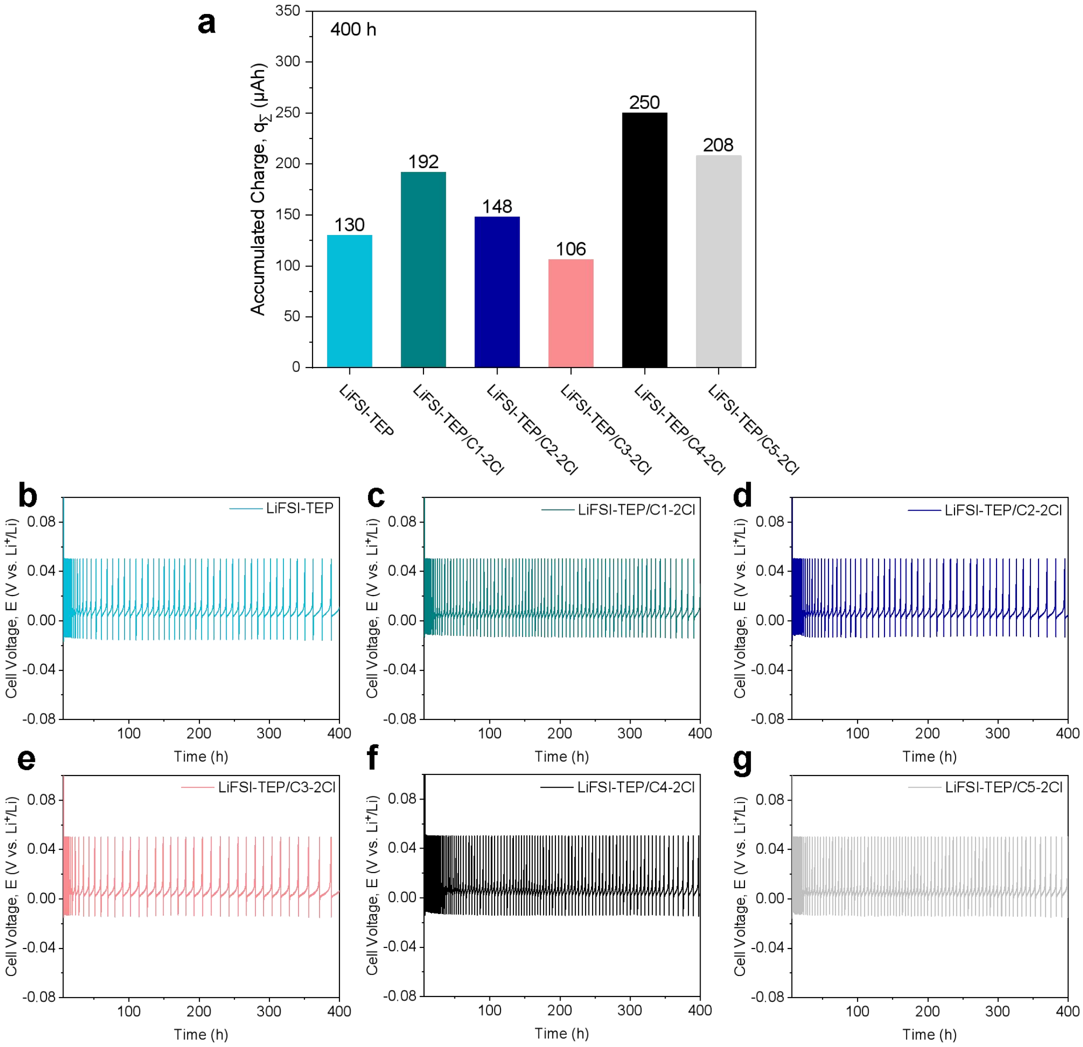

Supplementary Fig. 11 | a, Accumulated charges of Li||Cu cells with different electrolytes. Voltage-time curves of Li||Cu cells with **b**, LiFSI-TEP, **c**, LiFSI-TEP/C1-2Cl, **d**, LiFSI-TEP/C2-2Cl, **e**, LiFSI-TEP/C3-2Cl, **f**, LiFSI-TEP/C4-2Cl, **g**, LiFSI-TEP/C5-2Cl.

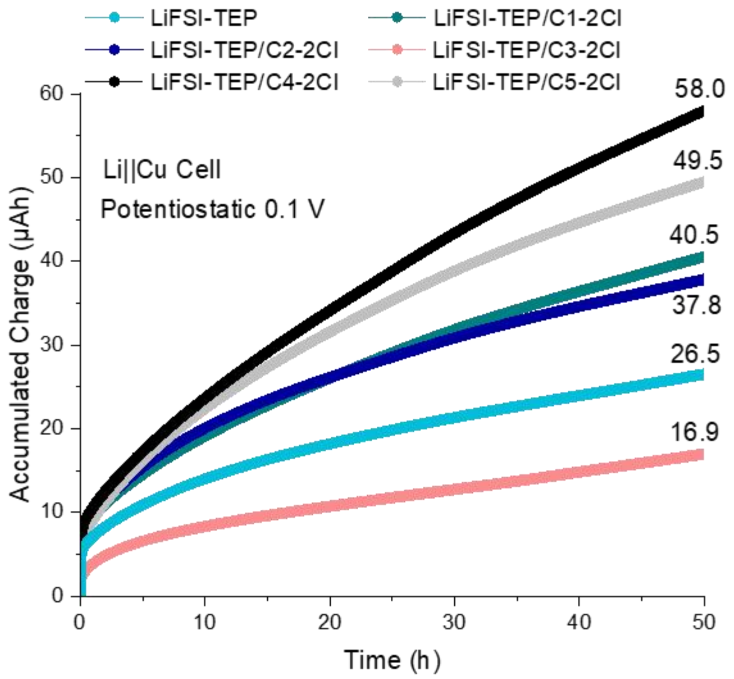

Supplementary Fig. 12 | Accumulated charges of Li||Cu cells with different electrolytes at a 0.1 V potentiostatic holding for 50 h.

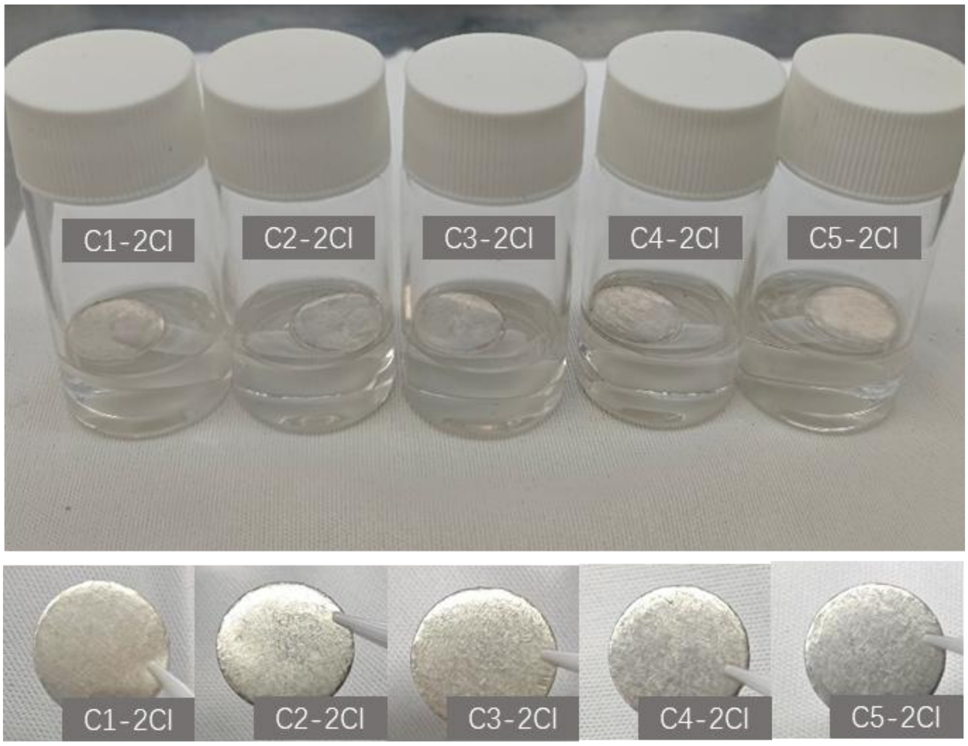

Supplementary Fig. 13 | Optical images of the state of Li metal sheets soaked in different diluents after 2 weeks.

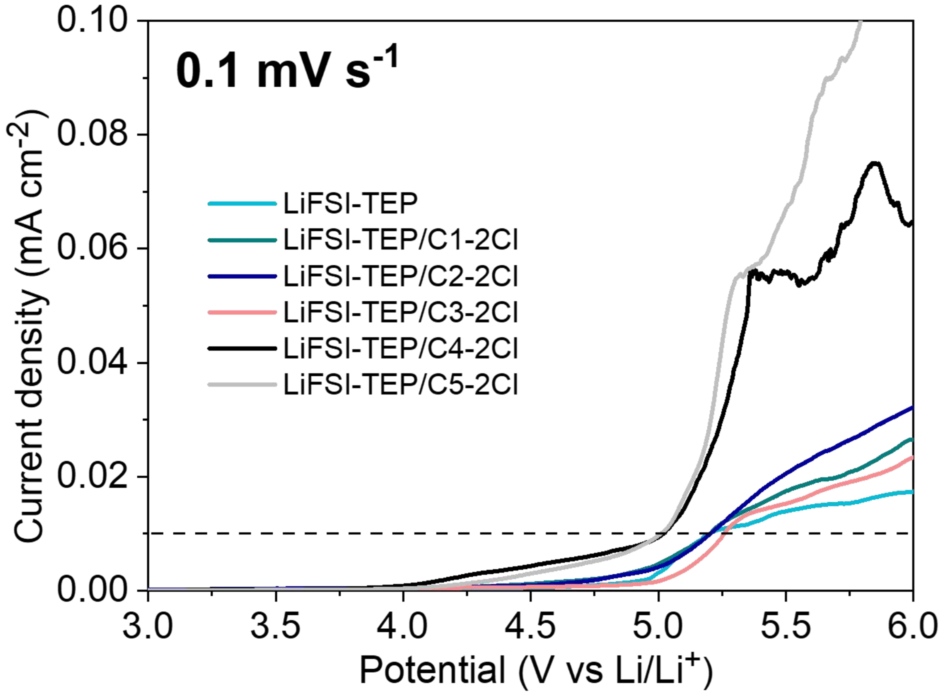

Supplementary Fig. 14 | LSV curves of Li||Al cells with different electrolytes under a scanning rate of 0.1 mV s^{-1}.

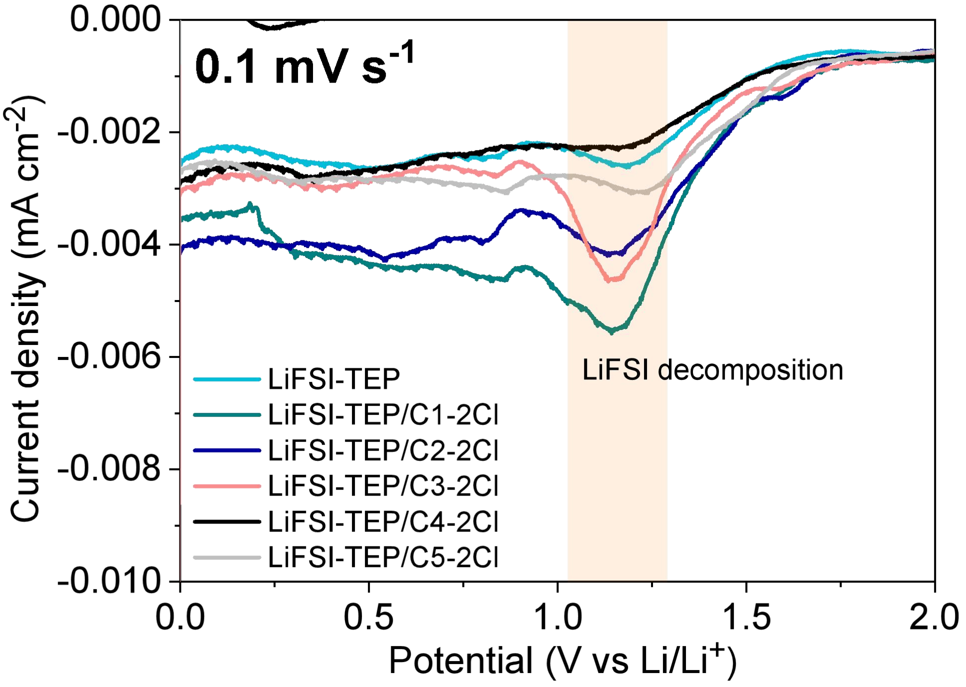

Supplementary Fig. 15 | Enlarged CV curves of Li||Cu cells with different electrolytes from 2.0 V to 0 V.

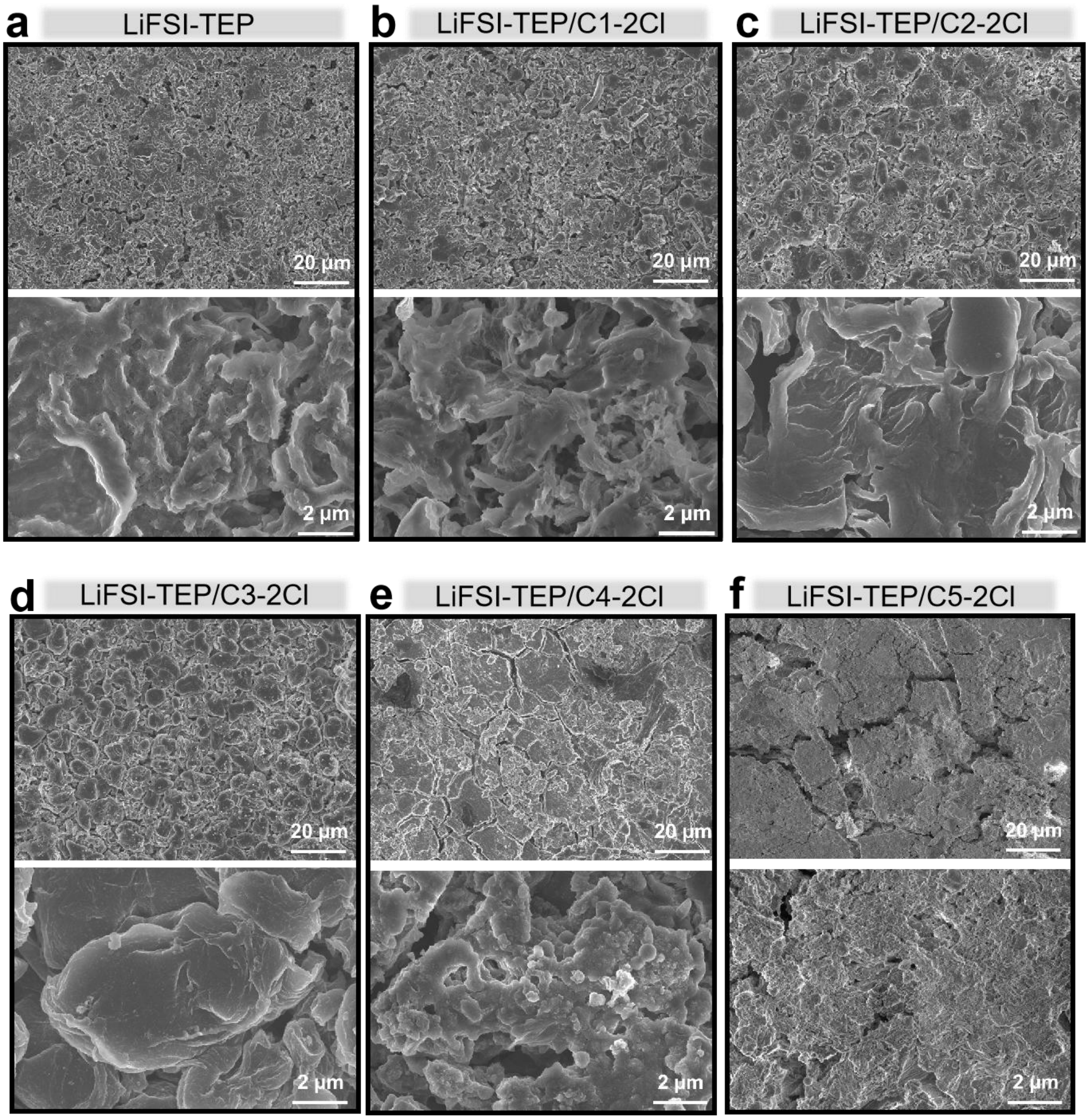

Supplementary Fig. 16 | SEM images of Li metal electrode surfaces after 50 cycles in different electrolytes. **a**, LiFSI-TEP. **b**, LiFSI-TEP/C1-2Cl. **c**, LiFSI-TEP/C2-2Cl. **d**, LiFSI-TEP/C3-2Cl. **e**, LiFSI-TEP/C4-2Cl. **f**, LiFSI-TEP/C5-2Cl.

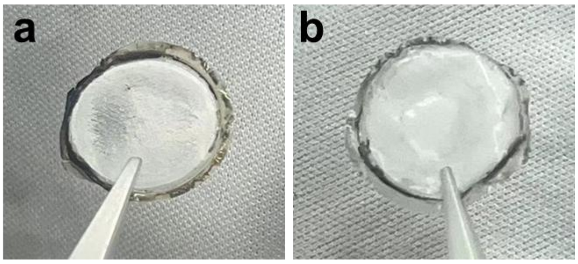

Supplementary Fig. 17 | Optical images of the polypropylene separators assembled from the Li||Li symmetric cells after 50 cycles in electrolytes. **a**, LiFSI-TEP/C4-2Cl. **b**, LiFSI-TEP/C5-2Cl.

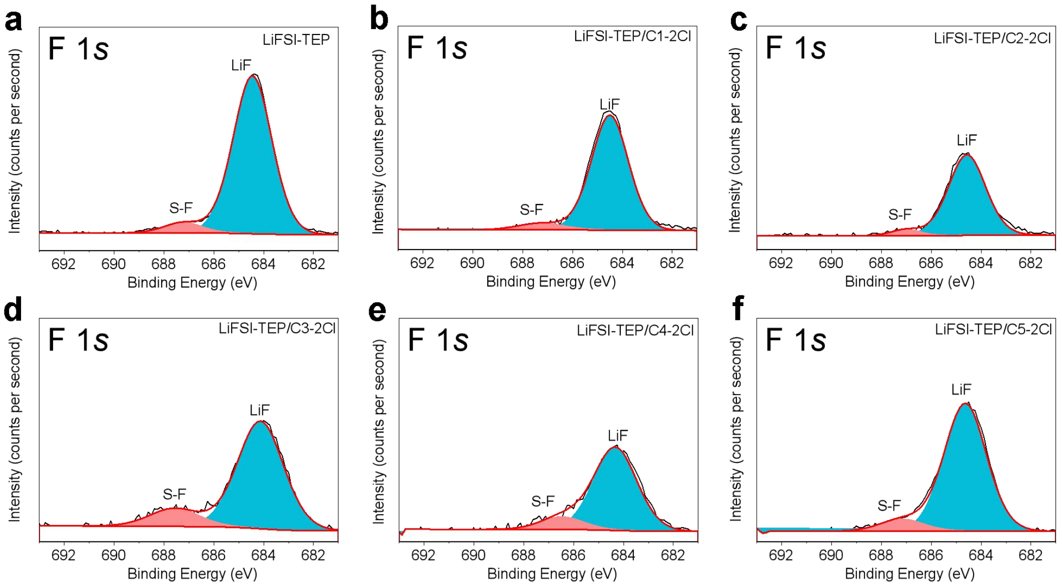

Supplementary Fig. 18 | F 1*s* XPS spectra of the SEI layers formed on the Li metal electrode surfaces after 50 cycles in different electrolytes.

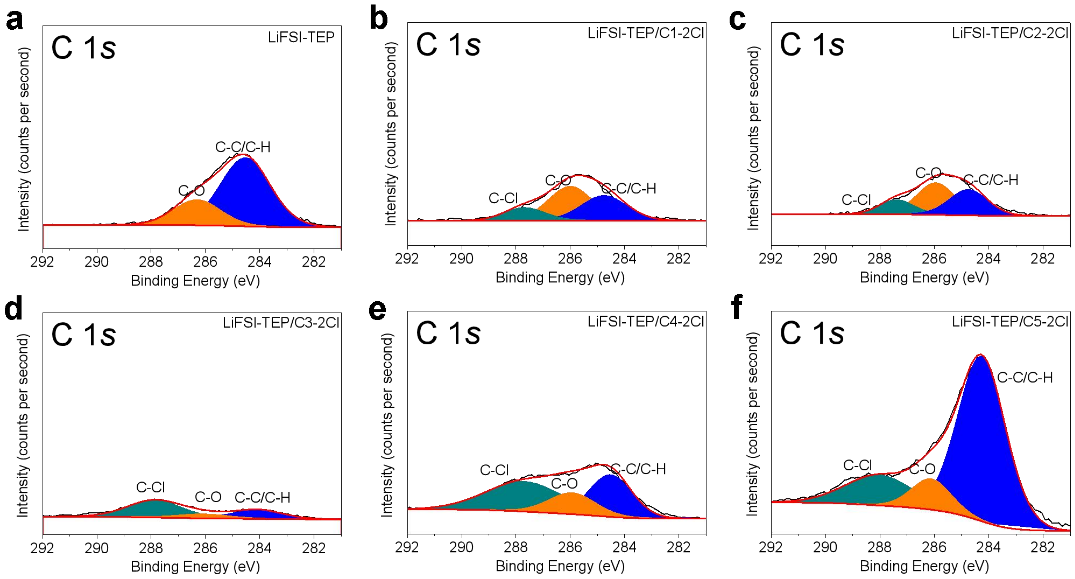

Supplementary Fig. 19 | C 1*s* XPS spectra of the SEI layers formed on the Li metal electrode surfaces after 50 cycles in different electrolytes.

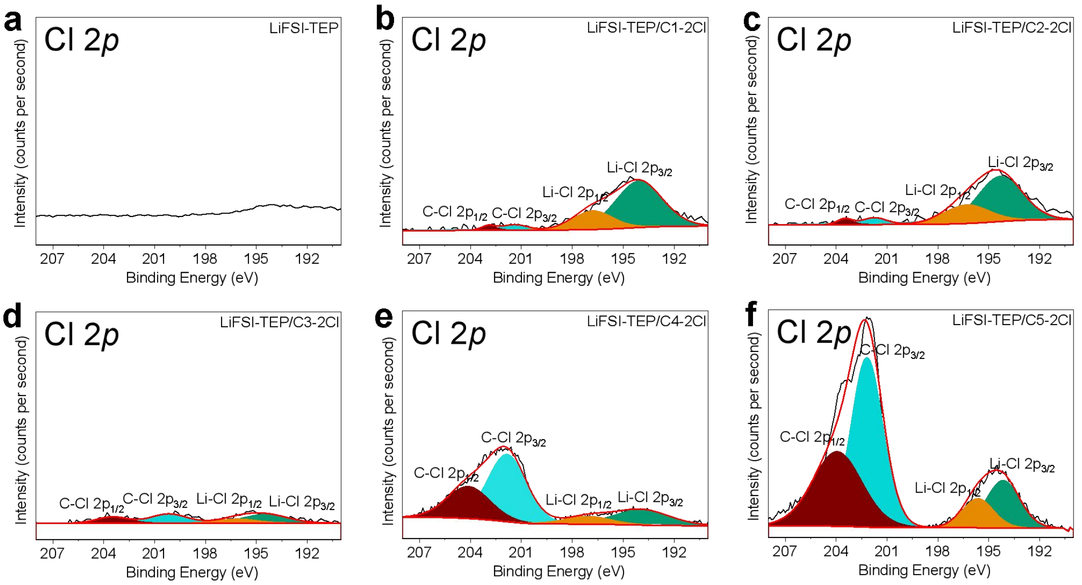

Supplementary Fig. 20 | Cl 2*p* XPS spectra of the SEI layers formed on the Li metal electrode surfaces after 50 cycles in different electrolytes.

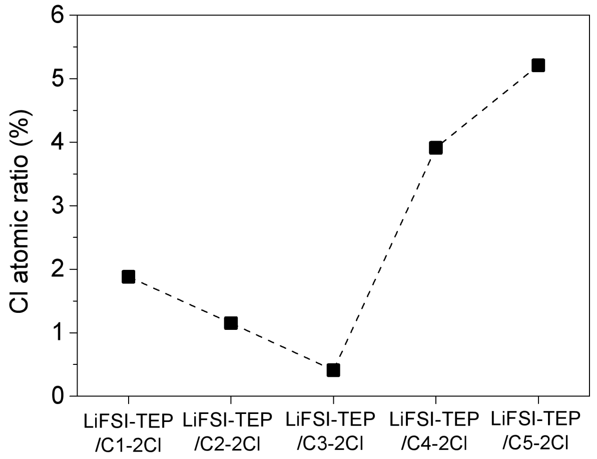

Supplementary Fig. 21 | Cl atomic ratios obtained by XPS analysis in the SEI layers formed on the Li metal surface with different electrolytes.

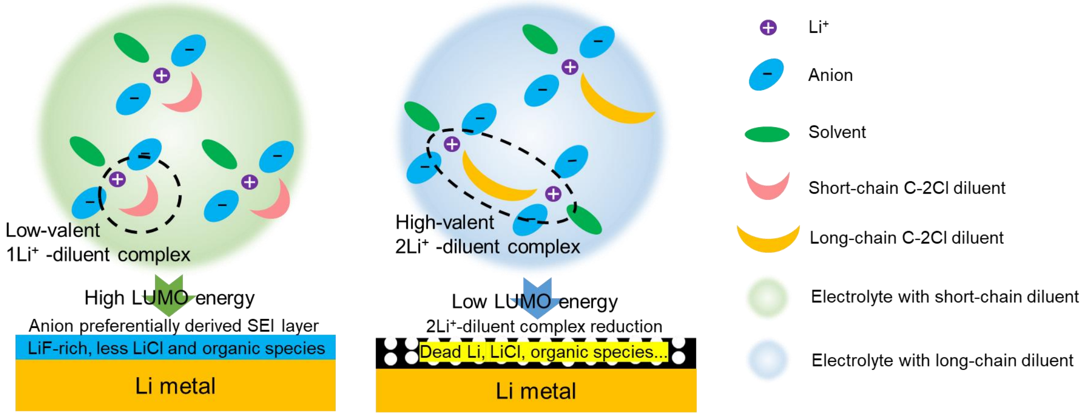

Supplementary Fig. 22 | Mechanism diagram of the solvation structure and SEI chemistry in electrolytes with short-chain C-2Cl diluent or long-chain C-2Cl diluent.

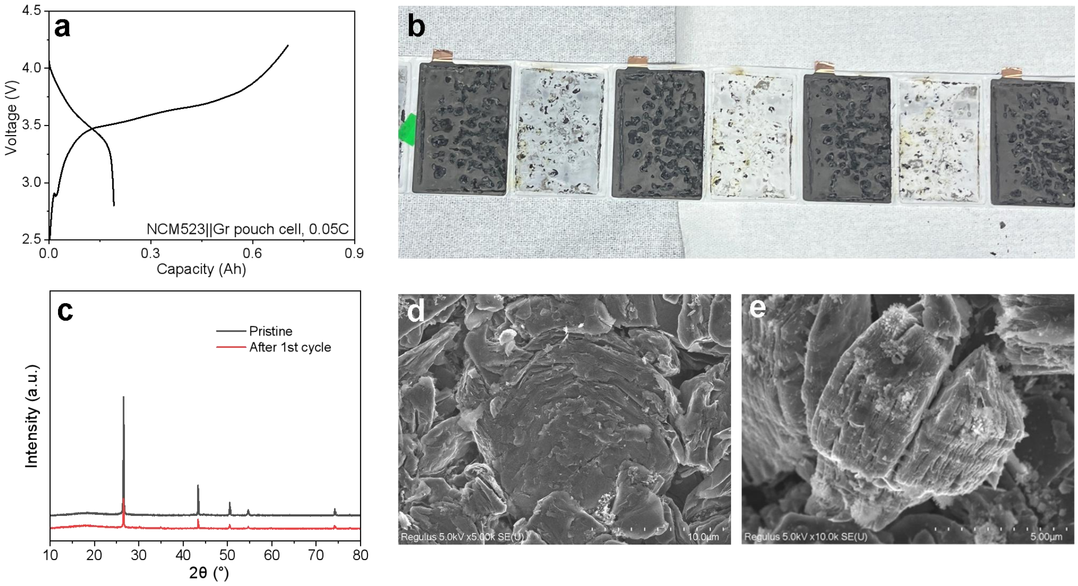

Supplementary Fig. 23 | Study on LiFSI-TEP/C3-2Cl electrolyte in NCM523||Gr pouch cell. **a**, Initial charge-discharge profile of pouch cell at 0.05C. **b**, Optical image of cycled Gr anodes and separators assembled from pouch cell. **c**, XRD spectra of pristine and cycled Gr electrodes. SEM images of the morphology of **d**, pristine Gr and **e**, cycled Gr.

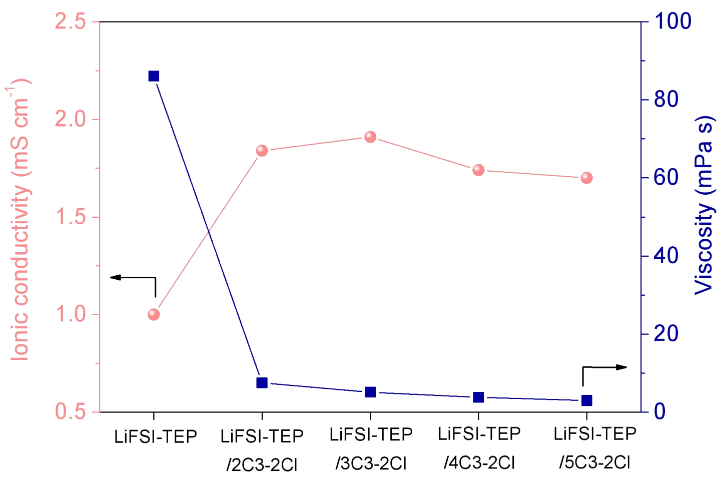

Supplementary Fig. 24 | Ionic conductivity and viscosity tests of electrolytes with different ratios of C3-2Cl diluent.

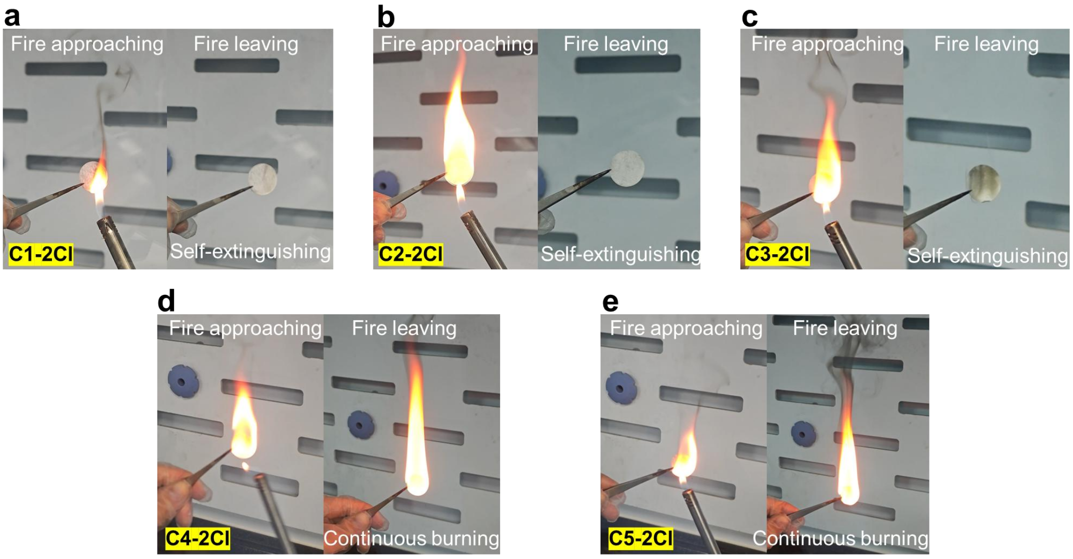

Supplementary Fig. 25 | Combustion tests of C-2Cl diluents with different chain-length. **a**, C1-2Cl, **b**, C2-2Cl, **c**, C3-2Cl, **d**, C4-2Cl, **e**, C5-2Cl.

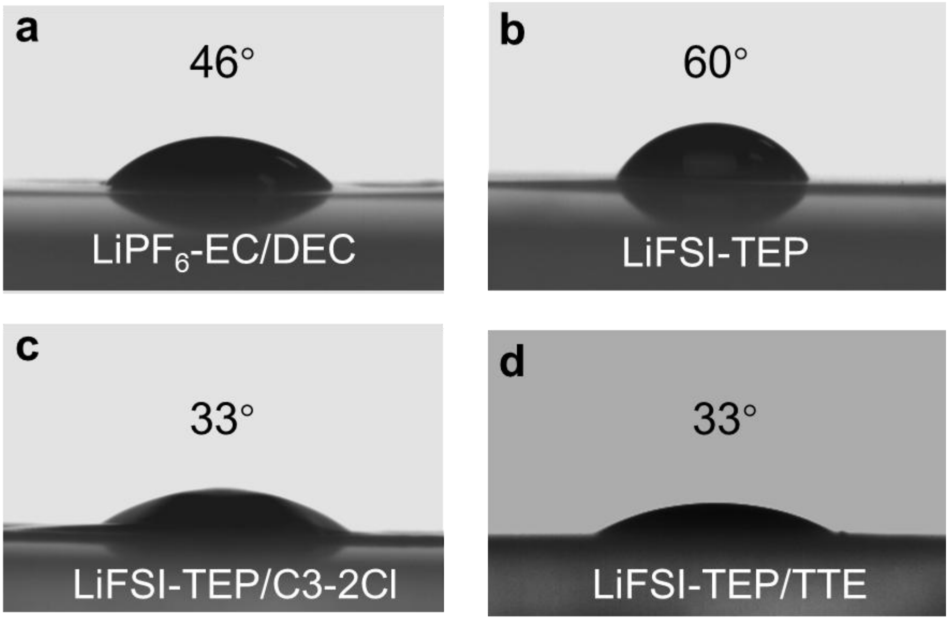

Supplementary Fig. 26 | Contact angle tests of different electrolytes. **a**, $LiPF_6$-EC/DEC, **b**, LiFSI-TEP, **c**, LiFSI-TEP/C3-2Cl, **d**, LiFSI-TEP/TTE.

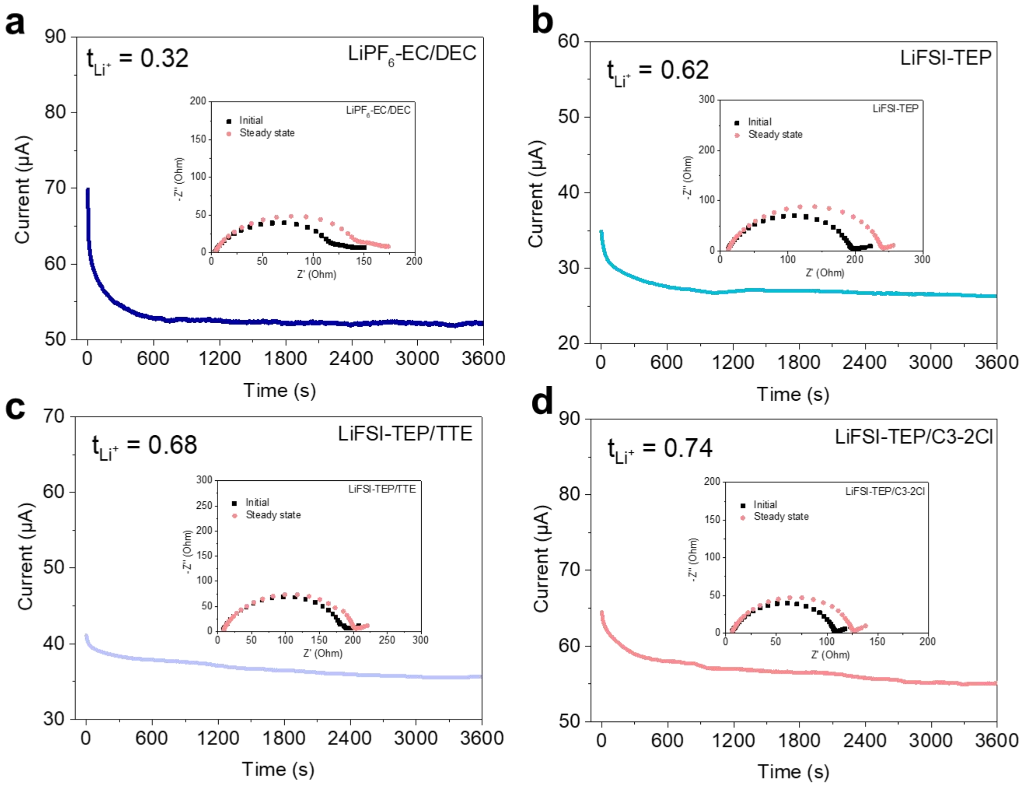

Supplementary Fig. 27 | Li^+ transference number measurements of different electrolytes. **a**, $LiPF_6$-EC/DEC. **b**, LiFSI-TEP. **c**, LiFSI-TEP/TTE. **d**, LiFSI-TEP/C3-2Cl.

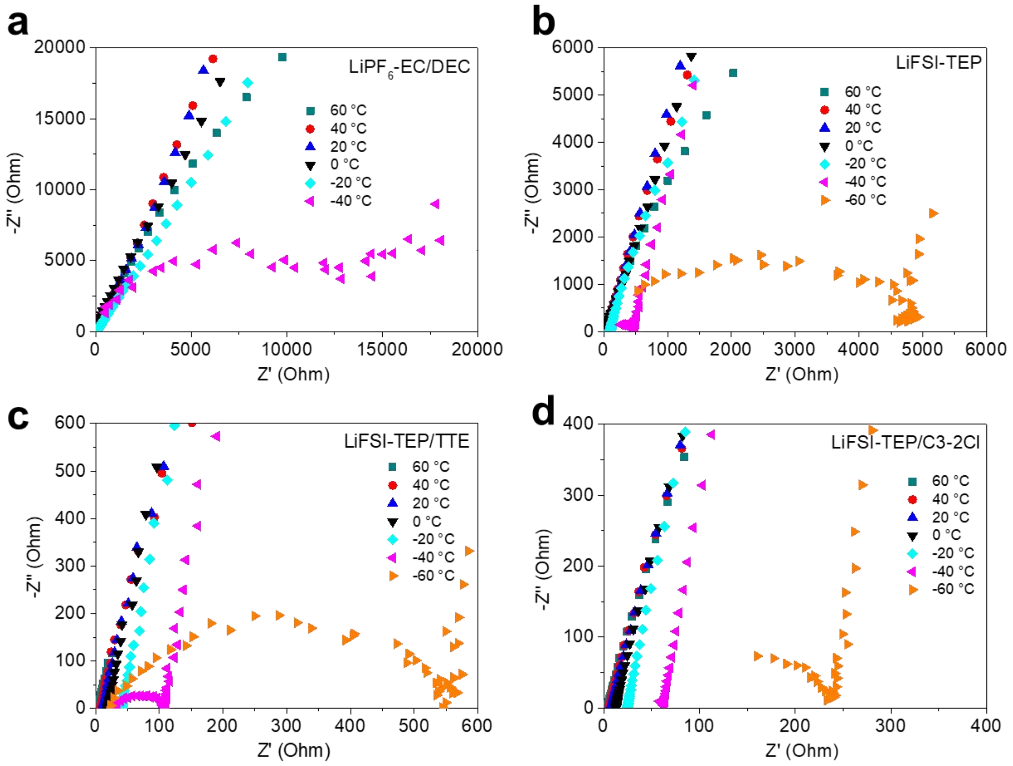

Supplementary Fig. 28 | EIS analysis of stainless steel (SS)||SS symmetric cells under different temperatures with different electrolytes. **a**, $LiPF_6$-EC/DEC. **b**, LiFSI-TEP. **c**, LiFSI-TEP/TTE. **d**, LiFSI-TEP/C3-2Cl.

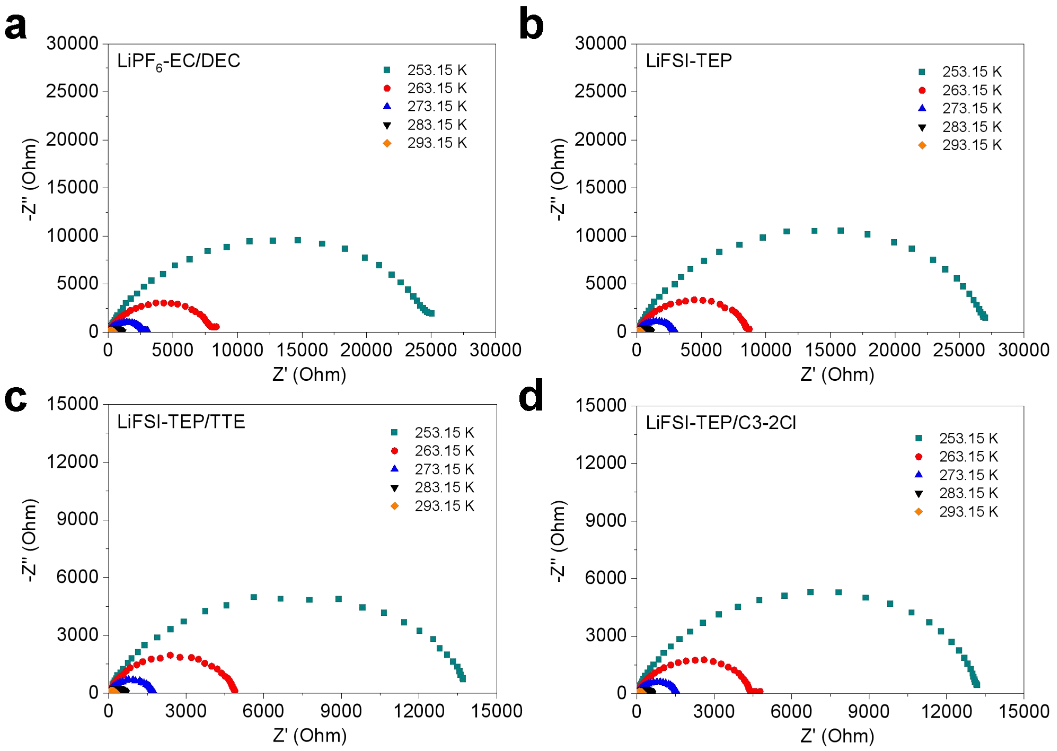

Supplementary Fig. 29 | EIS analysis of Li||Li symmetric cells at different temperatures with different electrolytes. **a**, $LiPF_6$-EC/DEC. **b**, LiFSI-TEP. **c**, LiFSI-TEP/TTE. **d**, LiFSI-TEP/C3-2Cl.

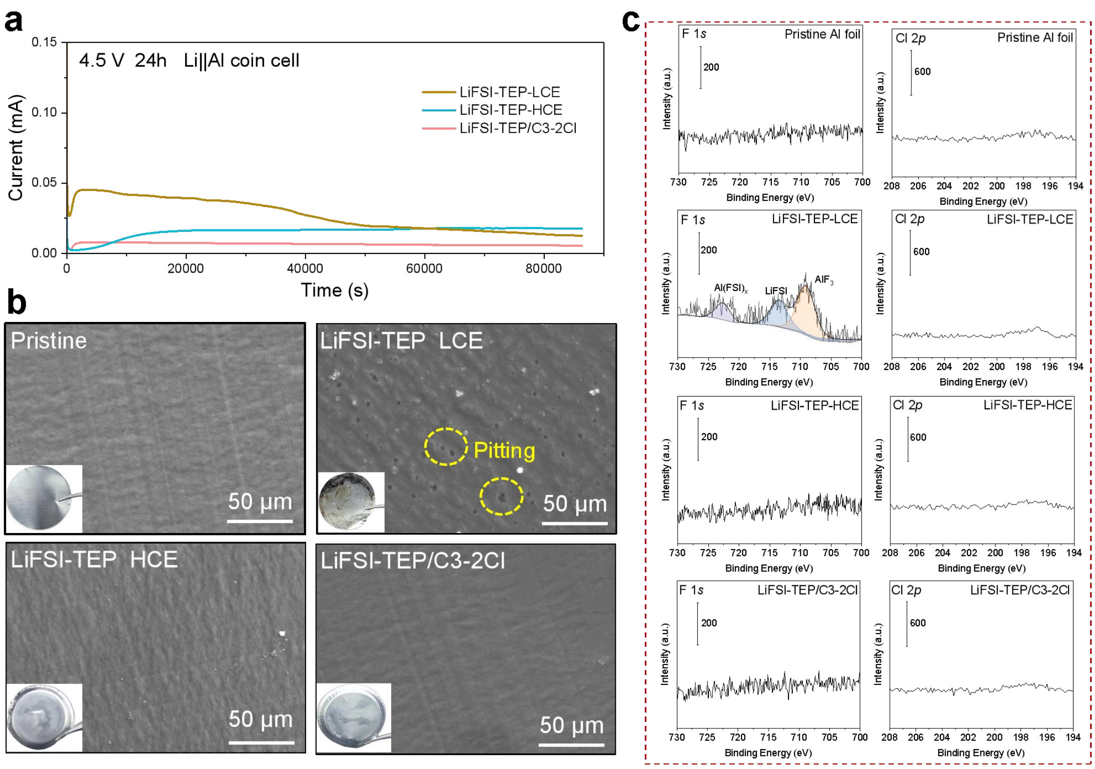

Supplementary Fig. 30 | Stability of Al current collectors in different electrolytes. **a**, Current-time curves of Li||Al coin cells with different electrolytes under 4.5 V constant voltage charging for 24h. **b**, SEM images of the morphologies of Al foils after charging in different electrolytes. **c**, F 1s and Cl 2*p* XPS spectra of the surface of Al foils after charging in different electrolytes.

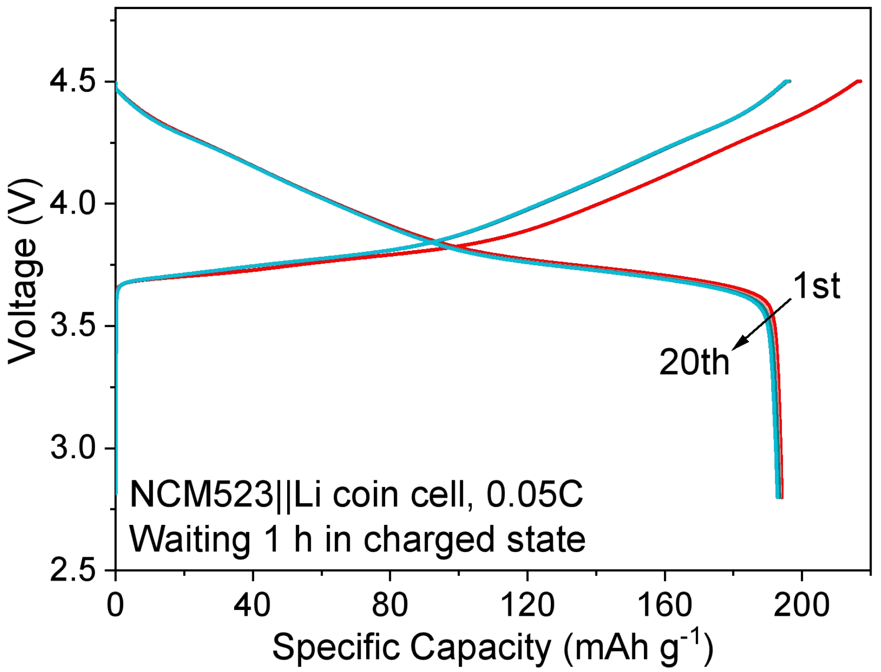

Supplementary Fig. 31 | Charge-discharge curves of Li||NCM523 coin cell with LiFSI-TEP/C3-2Cl at initial 20 cycles with 0.05C rate and waiting 1 h in 4.5 V charged state.

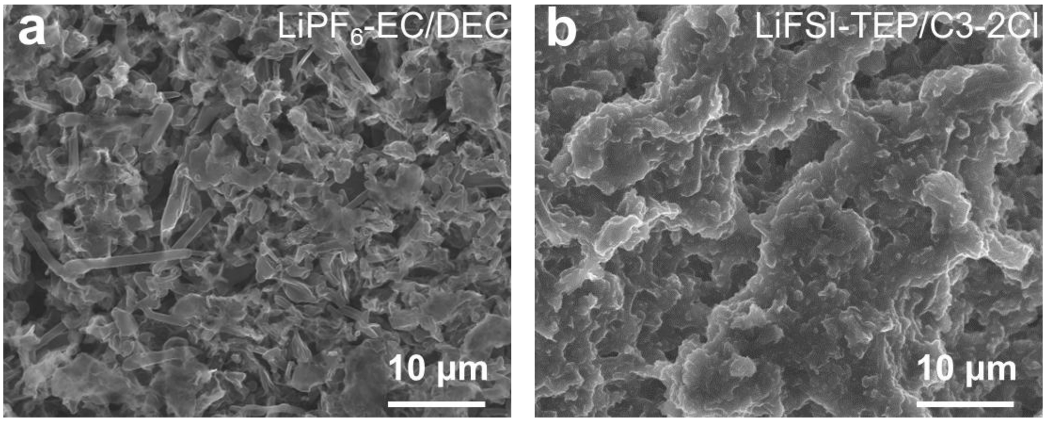

Supplementary Fig. 32 | SEM images of the surface morphology of Li metal electrode after 50 cycles in different electrolytes. **a**, $LiPF_6$-EC/DEC and **b**, LiFSI-TEP/C3-2Cl.

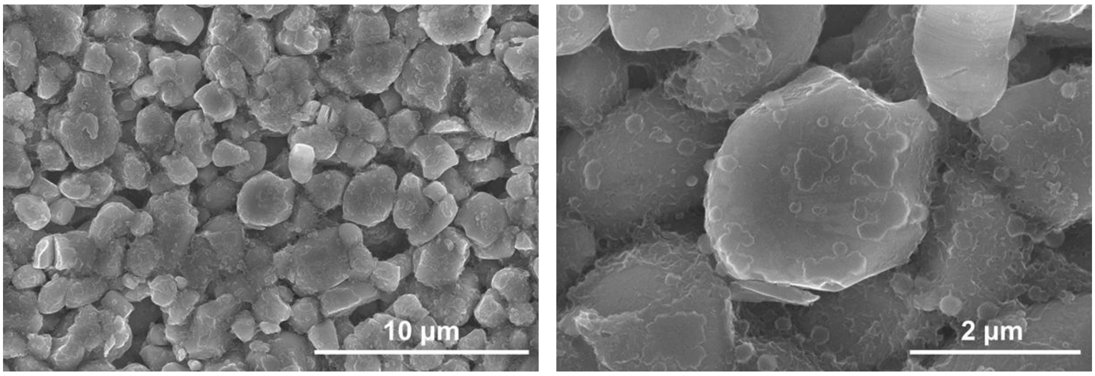

Supplementary Fig. 33 | SEM images of the surface morphology of NCM83 electrode after 50 cycles in LiFSI-TEP/C3-2Cl.

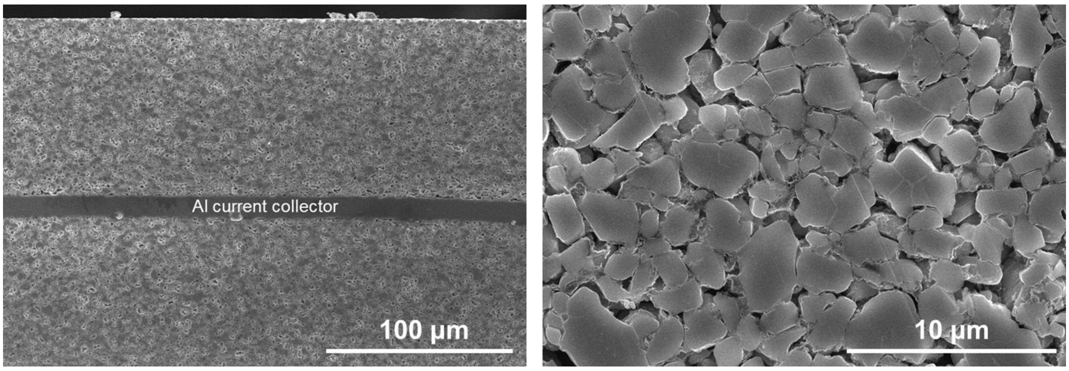

Supplementary Fig. 34 | SEM images of the cross-sectional morphology of NCM83 electrode after 50 cycles in LiFSI-TEP/C3-2Cl.

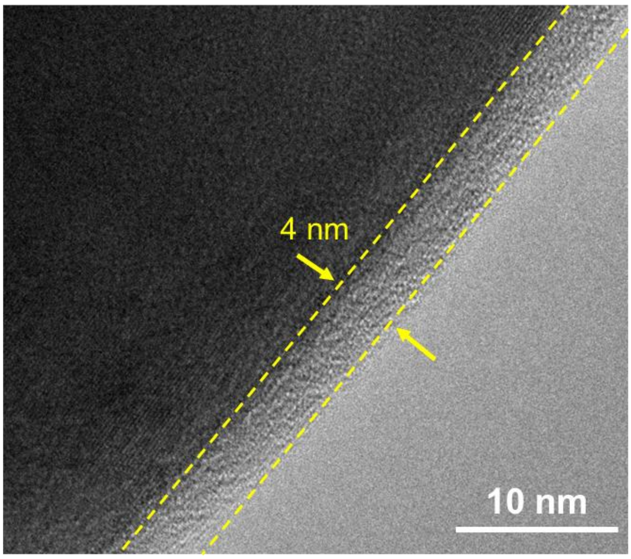

Supplementary Fig. 35 | TEM image of the thickness of CEI layer formed on the surface of NCM83 particle after 50 cycles in LiFSI-TEP/C3-2Cl.

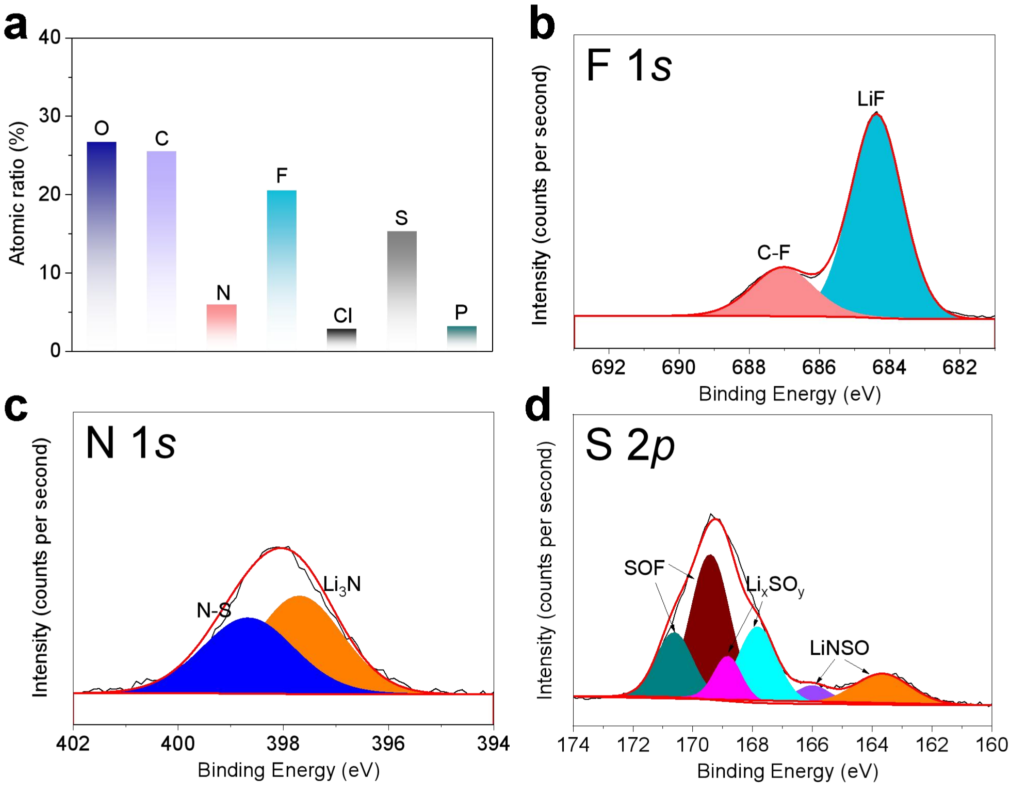

Supplementary Fig. 36 | XPS analysis of the components of CEI layer formed on the surface of NCM83 particle after 50 cycles in LiFSI-TEP/C3-2Cl. **a**, Atomic ratio analysis. **b**, F 1*s* spectrum. **c**, N 1*s* spectrum. **d**, S 2*p* spectrum.

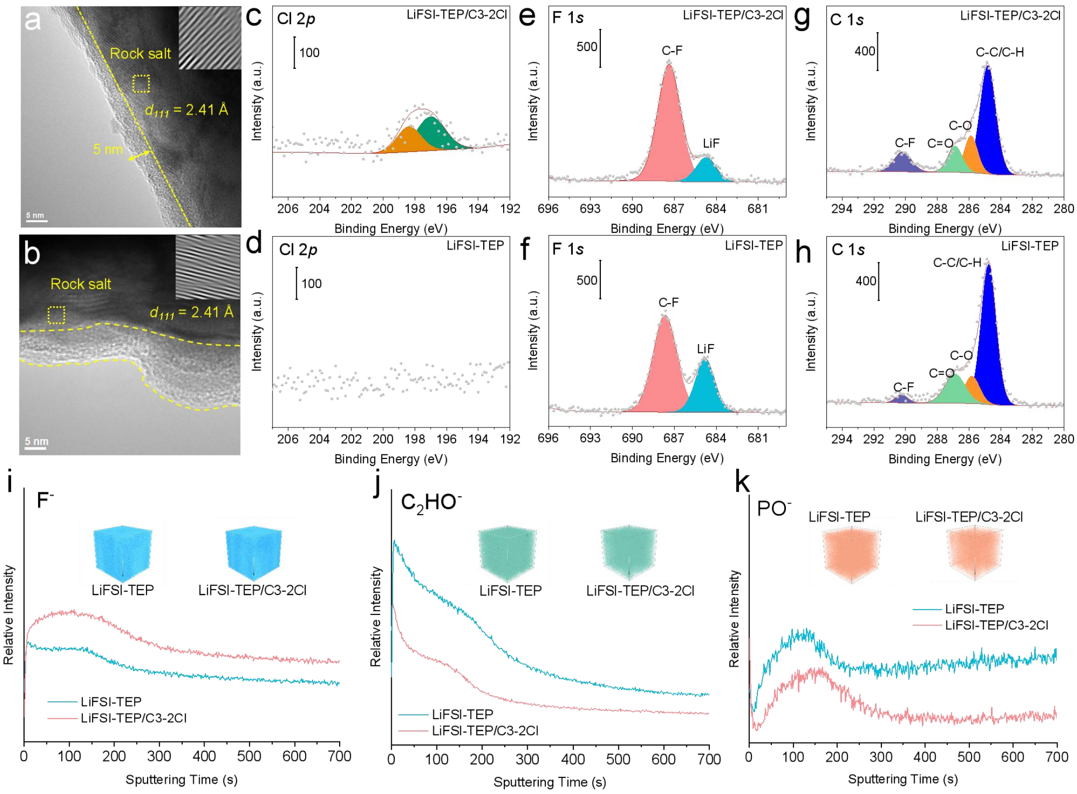

Supplementary Fig. 37 | Characterizations of CEI layers formed on the 4.5 V NCM523 electrodes after 50 cycles. TEM of CEI layers formed by **a**, LiFSI-TEP/C3-2Cl and **b**, LiFSI-TEP. XPS spectra of CEI layers formed by **c**, **e**, **g**, LiFSI-TEP/C3-2Cl and **d**, **f**, **h**, LiFSI-TEP. TOF-SIMS of **i**, F^-, **j**, C_2HO^- and **k**, PO^- species in CEI layers formed by different electrolytes.

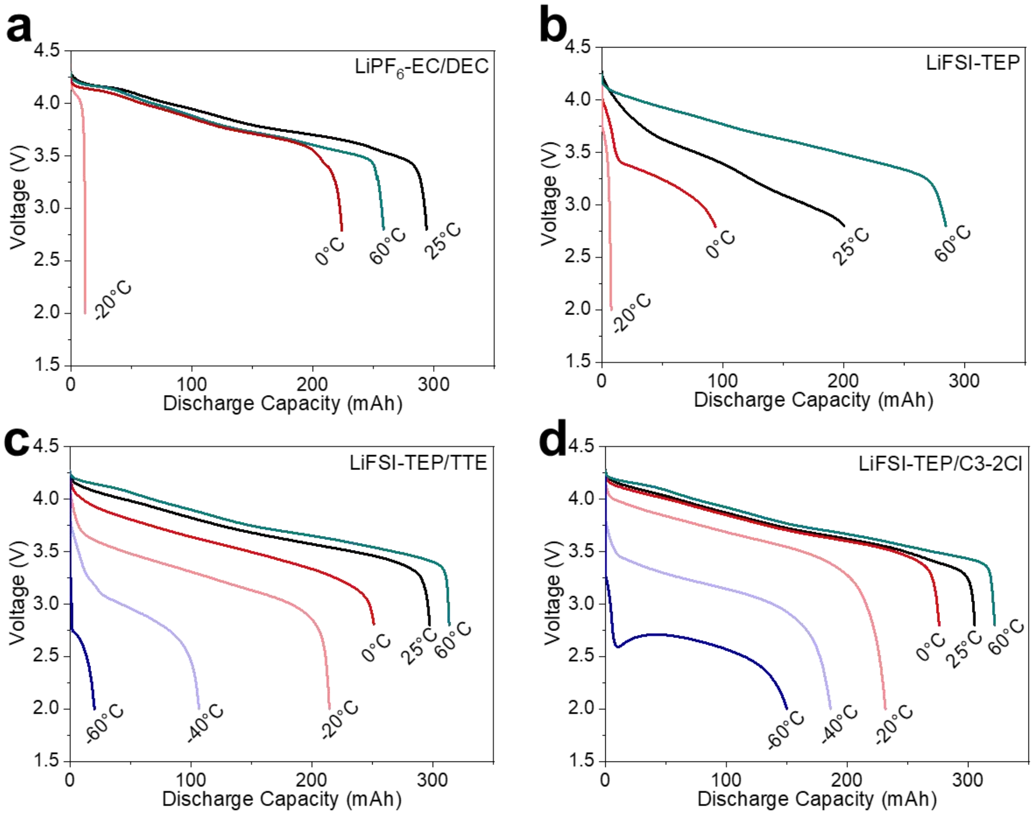

Supplementary Fig. 38 | Discharge capacities of Li||NCM83 pouch cell with different electrolytes at different temperatures. **a**, $LiPF_6$-EC/DEC. **b**, LiFSI-TEP. **c**, LiFSI-TEP/TTE. **d**, LiFSI-TEP/C3-2Cl.

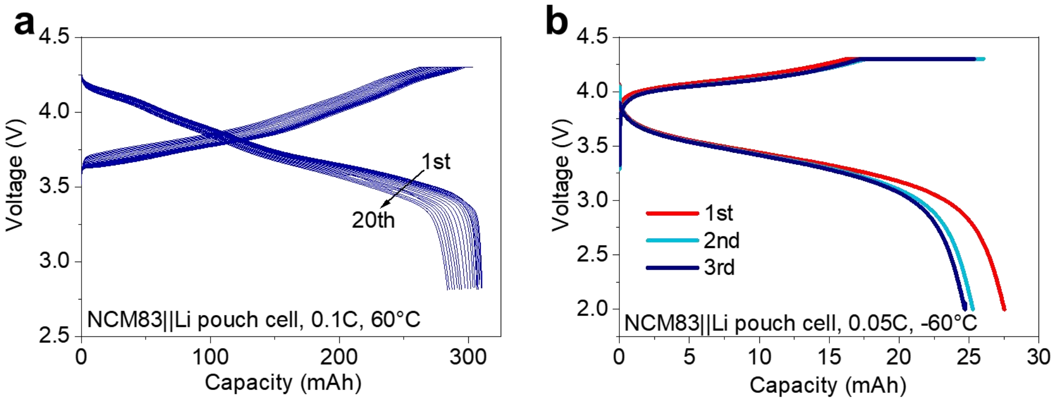

Supplementary Fig. 39 | a, 60 ℃ high-temperature and **b**, -60 ℃ low-temperature cycling performance of Li||NCM83 pouch cell with LiFSI-TEP/C3-2Cl.

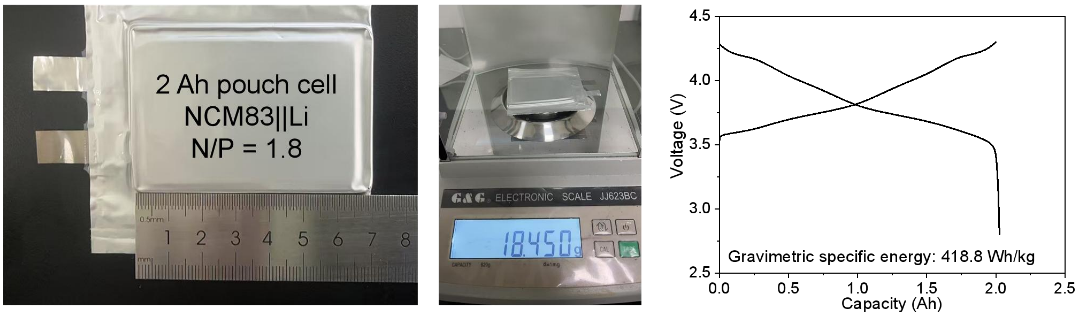

Supplementary Fig. 40 | Optical image, overall weight and 0.05C charge-discharge profiles of 2 Ah-level pouch cell with LiFSI-TEP/C3-2Cl.

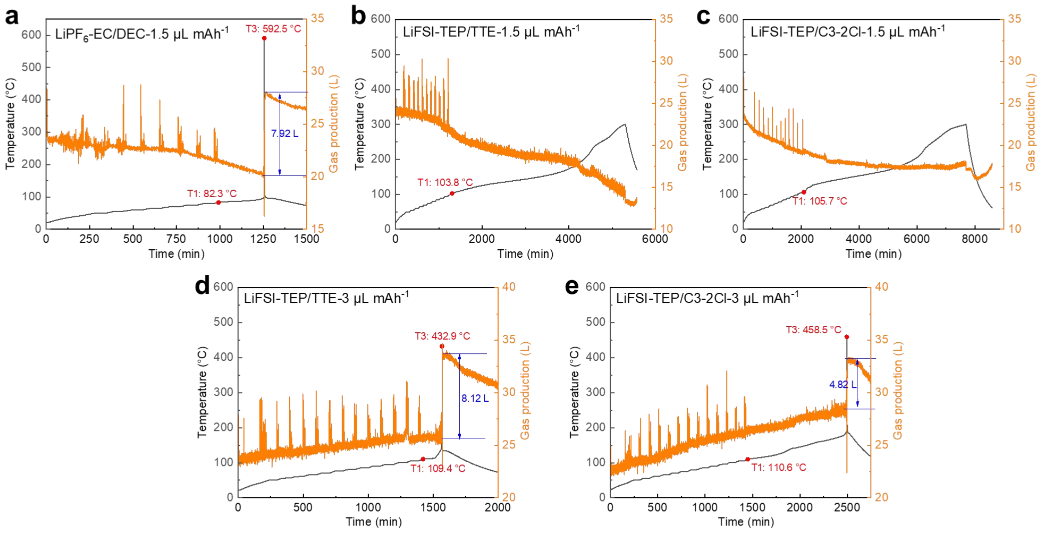

Supplementary Fig. 41 | ARC results with temperature-time and gas production-time curves of Li||NCM83 pouch cells in **a**, $LiPF_6$-EC/DEC with an electrolyte amount of 1.5 μL mAh^{-1}, **b**, LiFSI-TEP/TTE with an electrolyte amount of 1.5 μL mAh^{-1}, **c**, LiFSI-TEP/C3-2Cl with an electrolyte amount of 1.5 μL mAh^{-1}. **d**, LiFSI-TEP/TTE with an electrolyte amount of 3 μL mAh^{-1}, **e**, LiFSI-TEP/C3-2Cl with an electrolyte amount of 3 μL mAh^{-1}.

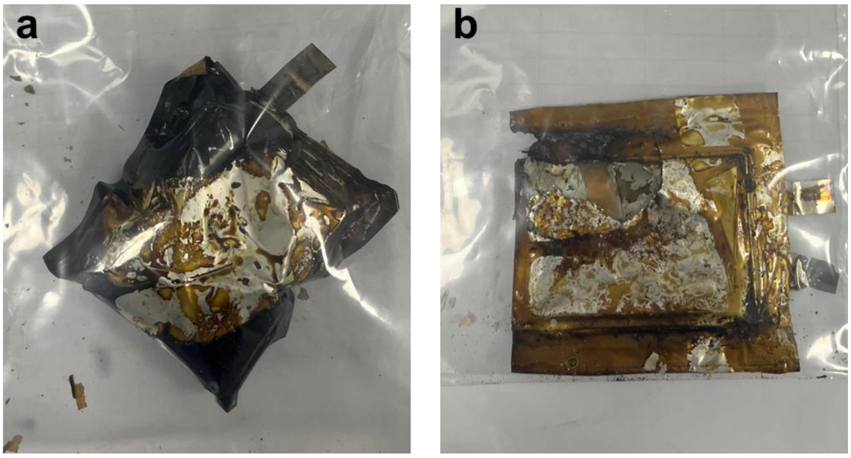

Supplementary Fig. 42 | Optical images of pouch cells after ARC test in **a**, LiFSI-TEP/TTE and **b**, LiFSI-TEP/C3-2Cl with an electrolyte amount of 1.5 μL mAh^{-1}.

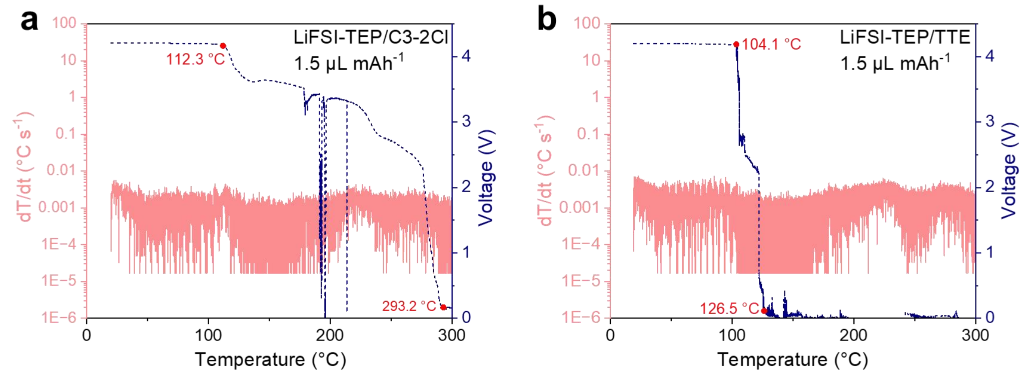

Supplementary Fig. 43 | ARC results with dT/dt-temperature and voltage-temperature curves of Li||NCM83 pouch cell in **a**, LiFSI-TEP/C3-2Cl and **b**, LiFSI-TEP/TTE with an electrolyte amount of 1.5 μL mAh^{-1}.

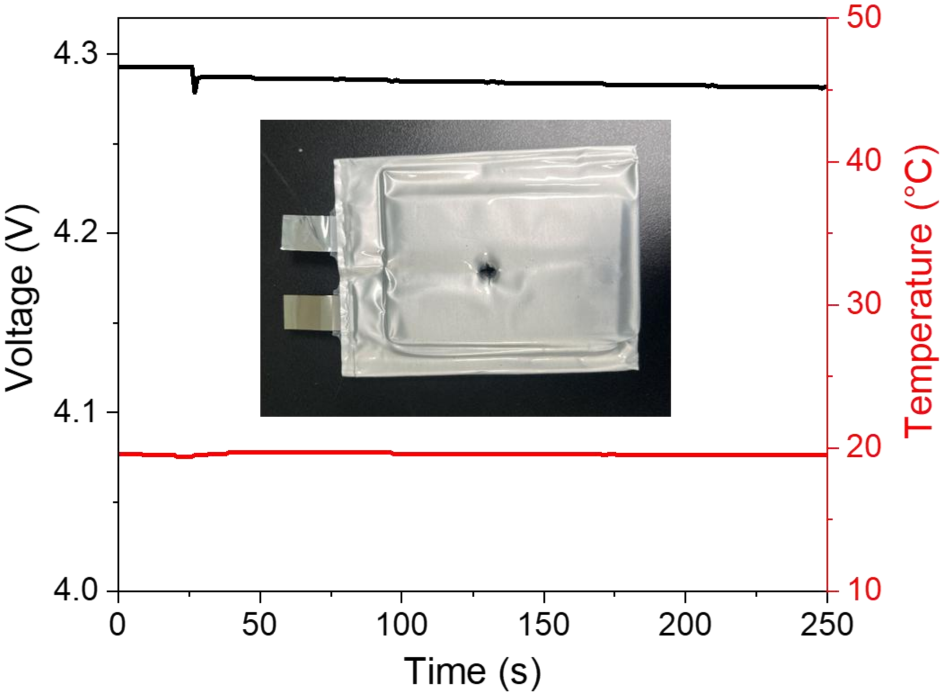

Supplementary Fig. 44 | Nail penetration short circuit test and post-test optical image of 2 Ah-level Li||NCM83 pouch cell with LiFSI-TEP/C3-2Cl.

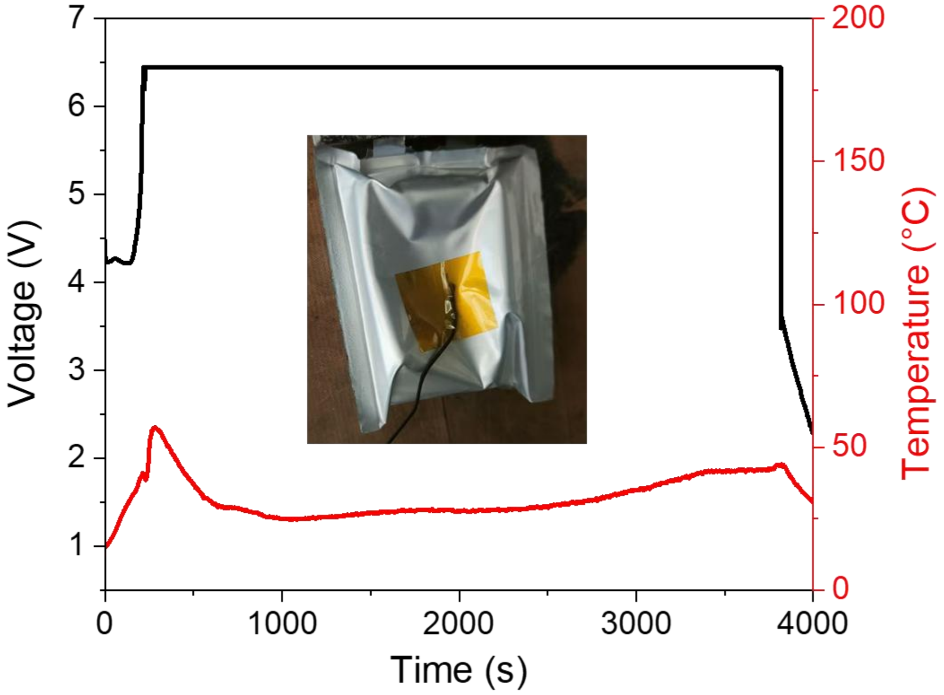

Supplementary Fig. 45 | Overcharge abuse test and post-test optical image of 2 Ah-level Li||NCM83 pouch cell with LiFSI-TEP/C3-2Cl.
