# Supplementary material for: Flame-retardant electrolytes with electrochemically-inert and weakly coordinating dichloroalkane diluents for practical lithium metal batteries
Source: Nat Commun. 2025 Nov 19;16:10188. doi: 10.1038/s41467-025-65138-8 (PMC12630897; doi:10.1038/s41467-025-65138-8)
Supplement: Supplementary file 2 — Description of Additional Supplementary Files [file 41467_2025_65138_MOESM2_ESM.pdf]

Description of Additional Supplementary Files

1. Supplementary Movie 1: Combustion test of $LiPF_6$-EC/DEC electrolyte.
2. Supplementary Movie 2: Combustion test of LiFSI-TEP/C3-2Cl electrolyte.
3. Supplementary Data 1: The source files of DFT calculations and MD structures.
